# Racing CARs to veterinary immuno-oncology

**DOI:** 10.3389/fvets.2023.1130182

**Published:** 2023-02-17

**Authors:** James R. Cockey, Cynthia A. Leifer

**Affiliations:** Department of Microbiology and Immunology, College of Veterinary Medicine, Cornell University, Ithaca, NY, United States

**Keywords:** applied immunology, cancer, cell therapy, immunotherapy, translational medicine

## Abstract

Chimeric antigen receptors (CARs) have demonstrated remarkable promise in human oncology over the past two decades, yet similar strategies in veterinary medicine are still in development. CARs are synthetically engineered proteins comprised of a specific antigen-binding single chain variable fragment (ScFv) fused to the signaling domain of a T cell receptor and co-receptors. Patient T cells engineered to express a CAR are directed to recognize and kill target cells, most commonly hematological malignancies. The U.S Food and Drug Administration (FDA) has approved multiple human CAR T therapies, but translation of these therapies into veterinary medicine faces many challenges. In this review, we discuss considerations for veterinary use including CAR design and cell carrier choice, and discuss the future promise of translating CAR therapy into veterinary oncology.

## 1. Introduction

Cell-based immunotherapy has progressed exponentially over the past few decades as a cutting-edge treatment option for multiple cancers. Adoptive cell therapy (ACT) involves harvesting immune cells from the patient, expanding them under good manufacturing practice (GMP) conditions, and reinfusing a clinically relevant dose. One of the first human ACTs used isolated tumor infiltrating lymphocytes (TILs) and selected for cells with a T cell receptor (TCR) specific toward a tumor neoantigen presented on MHC I of the tumor (1–3). Although promising (4–6), a significant advance in ACT that takes advantage of the specificity and affinity of antibodies against a tumor surface antigen, rather than relying on endogenous T cell receptors (TCRs), is chimeric antigen receptors (CARs). The FDA has approved multiple CAR T therapies against human B cell maturation antigen expressed on antibody-secreting plasma cells (7, 8), and CD19, which is expressed on the surface of almost all B cells (9–12). Similar to humans, lymphomas are common in companion animals. Retrospective analysis of 171 canine and feline non-Hodgkin's lymphoma samples revealed 79.9% of canine cases were B cell lymphomas that were predominantly multicentric, while 64.6% of feline cases were T cell lymphomas that were predominantly alimentary (13). While chemotherapy remains the standard of care in veterinary medicine (14), CARs are an attractive alternative or add on therapy for refractory veterinary lymphomas. Clinical trials have only recently been initiated in dogs. In this review, we outline the design of CARs and the future outlook of the therapy for veterinary use.

## 2. CAR construct design

**Figure 1:**
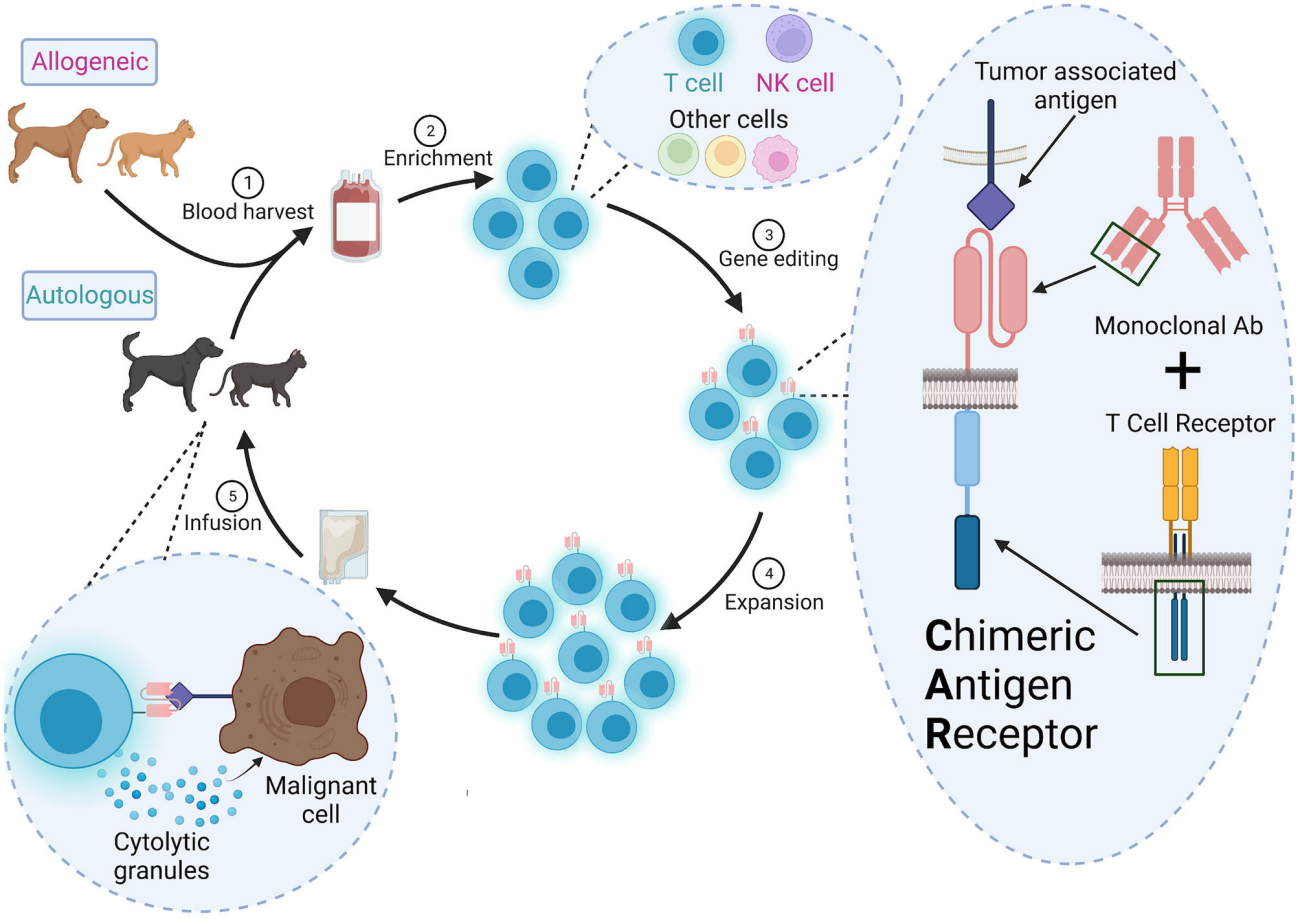
Overall scheme for CAR therapy in veterinary medicine. (1) Autologous (from the patient) or allogeneic (from a donor) cells are harvested from peripheral blood or apharesis, (2) enriched and (3) engineered to a express a CAR *ex vivo*. The CAR contains the variable heavy and light chains of a monoclonal antibody specific for a tumor-associated antigen and signaling domains from the TCR signaling complex. (4) The CAR^+^ cells are expanded to a clinically relevant dose and (5) infused into the patient. These CAR cells will detect and destroy cells expressing the target antigen.

Development of a CAR therapy requires multiple steps, each of which presents unique challenges for translation to veterinary medicine (Figure 1). In this section, we summarize basic CAR design and methods of expressing the CAR in primary cells.

CARs are created by stitching together an ScFv, a hinge, a transmembrane domain, and one or more cytoplasmic signaling domain(s) derived from the TCR signaling complex (15, 16). ScFvs are developed from the variable light and heavy chains of a specific monoclonal antibody targeting a tumor-associated antigen. Some CAR approaches use endogenous ligands or receptors, rather than ScFvs, to target tumors and may be a good alternative when cross-reactive or veterinary-specific antibodies are not available (17). Newer high-throughput fluorescence-activated cell sorting (FACS) screens can also be used to identify potential antibodies or ScFvs (18), but it is unclear if this strategy would be practical for clinical manufacturing in veterinary medicine.

The cytoplasmic signaling domains are critical to drive T cell activation and can lead to different effector functions in the patient. Use of one signaling domain, CD3ζ, resulted in low-level signaling, and poor persistence or anergy in patients (19, 20). CAR T therapies approved for human use have additional costimulatory receptor signaling domains like 4-1BB (Kymriah^®^, Breyanzi^®^, Abecma^®^, and Carvykti™) or CD28 (Yescarta^®^, Tecartus^®^). Human primary T cells transduced with a CAR containing the CD28 signling domain preferentially generated effector memory T cells *in vitro* (CCR7^−^CD45RO^+^) while the 4-1BB signlaing domain drove a central memory phenotype (CCR7^+^CD45RO^+^) (21). Using NSG mice with a xenografted osteosarcoma, infused human CAR T cells with 4-1BB had lower expression of exhaustion markers than those with CD28 (22). Some CARs use two costimulatory domains and have increased efficacy in preclinical animal models (23, 24). Comparison of efficacy of different CAR components in veterinary oncology remains limited and will likely require additional empirical testing (25).

CARs are frequenty delivered to patient primary T cells using a replication-incompetent lentivirus or γ-retrovirus (26, 27). Pre-activation is required because the viruses can only (γ-retrovirus), or preferentially (lentivirus), integrate into dividing cells (26, 27). However, other approaches have used transposons to integrate the CAR-encoding DNA (28, 29). To avoid delivery of viruses to patients, anti-canine CD20 CAR mRNA was directly electroporated into canine T cells (30, 31). However, CAR expression by mRNA delivery was transient and waned after 14 days (30). Lipid nanoparticles may enhance delivery of CAR mRNA and can be used *in vivo* (32). Transient CAR expression could be an advantage for veterinary therapy since it will limit immune reaction against the xenogeneic antibody components of the ScFv. Regardless of which CAR is developed, the sequences should be species-matched as much as possible to reduce host anti-CAR immune responses.

Gene editing tools like transcription activator-like effector nucleases (TALEN^®^) and clustered regularly interspaced palindromic repeats (CRISPR)/Cas9 allow for simultaneous delivery of the CAR and reduced graft-vs-host and host-vs-graft responses (33–36). For example, CAR insertion into the TCR locus allows for expression of the CAR under the endogenous transcriptional regulation of the TCR promoter, which limits exhaustion, and elimination of TCR expression, which reduces graft vs. host disease (37). Conversely, deletion of β2-microglobulin, part of MHC I, reduces CAR T cell rejection by the host. However, loss of MHC I increases detection and destruction by natural killer (NK) cells, which can be mitigated in part by knock-in of human leukocyte antigen E into the *B2M* locus (38). Inhibitory receptors such as PD-1, which limit CAR T cytotoxicity, can also be deleted using these gene editing tools (39). However, CRISPR can induce unwanted mutations (40, 41), or multiple donor DNA insertions (42). Unpredicted translocations have also occured when TALEN^®^ was used to delete the TCR alpha chain and CD52 to make “universal” CAR T cells (35). Fortunately, these off-target events are relatively rare (43).

## 3. Cell manufacturing

While GMP guidelines must be followed for clinical-stage ACT in both human and veterinary medicine, there are some specific considerations for veterinary application. In this section we will outline methods that are under investigation for veterinary CAR T cell expansion, as well as systems employed in human CAR T cell production that could be adapted for veterinary use.

Production of cells for clinical use requires validation of standard operating procedures and GMP-grade reagents and materials in all manufacturing steps with individual certificates of analysis. Growth of cells for human medicine requires serum-free, xeno-free, GMP-grade, commercial media formulations. There may not be commercially available GMP-grade species-specific sera for veterinary applications. Anti-canine CD20 CAR T cells failed to grow in OpTmizer™ serum-free media, and while there was some growth in LymphoONE™ serum-free media, CAR expression levels were low, suggesting empirical identification of optimal growth conditions for each veterinary CAR application may be necessary (44). Moreover, veterinary species cytokine supplements are limited (45) and thus validated cross-reactive reagents may be required (46). Feeder cells or special additives can enhance *ex vivo* expansion. For example, K562 cells can be engineered to express human CD32 and canine CD86, thereby acting as artificial antigen presenting cells (aAPCs). Co-culture of canine T cells with these aAPCs resulted in nearly six-fold expansion, and was even able to stimulate proliferation in T cells that were unresponsive to agonistic anti-canine CD3/CD28 beads (30). High CD8^+^ subset expansion and reduced PD-1/PD-L1 expression on canine CAR T cells occurred when the cells were grown with thyroid adenocarcinoma aAPCs expressing CD80, CD83, CD86, and 4-1BBL in the presence of phytohemagglutinin (47). Phytohemagglutinin also increased retroviral transduction efficiency (44). Additional advancements in cell culture using closed-system bioreactors can further enhance *ex vivo* expansion yield, reduce contamination risks, and minimize technician handling (26, 48–52). These devices will likely be employed more frequently in future veterinary clinical trials.

## 4. Choosing the right “CAR driver”

Currently all FDA approved human CAR therapies are T cell-based, and T cells are also the “driver” for canine CAR therapy. However, many different immune cells could potentially be used to carry a CAR (Table 1). In this section, we describe the major advantages and limitations of each cell type, as well as the development and therapeutic potential in veterinary medicine. The focus is on canine CARs since they have advanced the furtherest in veterinary medicine, but we will discuss potential use in felines and highlight findings in human medicine that have potential for veterinary applicability.

**Table 1:** Summary of species-specific surface markers that define immune cells and can be used to enrich desired populations through FACS or magnetic bead enrichment.

**Immune cell**	**Human phenotypic markers**	**Murine phenotypic markers**	**Canine phenotypic markers**	**Feline phenotypic markers**	**References**
T cell	CD3^+^CD56^−^αβTCR^+^	CD3^+^ αβTCR^+^NK1.1^−^	CD3^+^CD5^bright^NKp46^−^αβTCR^+^	CD3^+^CD56^−^αβTCR^+^	(53–58)
NK cell	CD3^−^CD56^+^CD7^+^	CD3^−^ NK1.1^+^ αβTCR^−^	CD3^−/+^ CD5^dim^CD8^+^TCRαβ^−^TCRγδ^−^ CD21^−^CD4^−^ CD94^+^ NKp46^+^	CD3^−^CD56^+^	(53, 54, 59–64)
NKT cell	CD3^+^CD56^+/−^iTCR^+^	CD3^+^NK1.1^+^iTCR^+^	CD3^+^CD5^intermediate^ NKp46^+^CD94^+^ iTCR^+^	CD3^+^CD56^+^	(53, 54, 59–61, 65–68)
γδ T cell	CD3^+^γδTCR^+^	CD3^+^γδTCR^+^	CD3^+^γδTCR^+^	CD3^+^γδTCR^+^	(69–71)
Macrophage	CD68^+^: CD80^+^CD206^dim^ (M1) or CD80^−^CD206^bright^ (M2)	F4/80^+^: CD38^+^ (M1) or CD38^−^ (M2)	Iba1^+^: CD204^−^ (M1) or CD204^+^ (M2)	Iba1^+^: CD204^−^(M1) or CD204^+^(M2)	(72–77)

### 4.1. T cells

The most advanced CAR therapeutics in veterinary medicine are T cells. The first clinical trial of CAR T cells in canine patients delivered a CD20 CAR mRNA by electroporation. The CAR contained a murine anti-canine CD20 ScFv with human CD8α leader, hinge, transmembrane, and a CD3ζ signaling domain (30). One canine patient with relapsed spontaneous B cell lymphoma was infused in three separate doses and had reduced CD20^+^ cell numbers with no adverse events. A follow up study treated diffuse large B cell lymphoma with anti-CD20 CAR containing the same ScFv, but canine signaling domains (78). No adverse events were documented following infusion in three dogs, but this therapy had lower efficacy and *in vivo* persistence of the cells was poor. Eventually, an escape-variant of CD20 was detected on peripheral blood B cells post-infusion. Additionally, two of the dogs developed anti-mouse ScFv CAR serum antibodies, which peaked at day 50 post-infusion. These types of anti-CAR immune responses can be reduced by generating a “caninized” ScFv where all but the complementarity determining regions of the ScFv are canine.

Preclinical and clinical investigation of canine CAR T cells has also begun to target solid tumors, which have notoriously been resistant in human CAR therapy. A HER2 CAR T cell therapy (79) with canine CD28 and CD3ζ signaling domains secreted IFNγ and was cytotoxic against HER2^+^ osteosarcoma and breast cancer target cell lines *in vitro* (80). IL13Rα canine CAR T cells secreted IFNγ when incubated with IL13Rα^+^ targets (81). A canine glioma cell line implanted into mouse brains was effectively eliminated using canine CAR T cells against IL13Rα with either a human or a canine 4-1BB signaling domain. B7-H3 CAR T cells (82) were more cytotoxic than HER2 CAR T cells toward canine osteosarcoma spheroids, but cytotoxicity was similar for the constructs incorporating CD28 or 4-1BB signaling domains (83). Two healthy canine subjects were then infused with either frozen or fresh autologous B7-H3 CAR T cells. The fresh infusion did induce a grade 2 toxicity but no other adverse events were observed, while the recipient of frozen cells had an allergic reaction 67 days later that was likely unrelated to the infusion. Together, these results show that canine CAR T cells are safe and well-tolerated, even for some solid tumors.

The most notable drawback of human CAR T therapy is cytokine release syndrome, which presents with pyrexia, delirium, hypotension, and increased serum IL6, and often requires administration of the IL6 receptor antagonist tocilizumab and steroids (84). To enhance safety and rapidly deplete infused cells in the event of an adverse reaction, drug-sensitive “kill switches” can be incorporated into the CAR (85–89). Since some adverse reactions have been observed in canine CAR T trials, including a case report of increased serum cytokines consistent with cytokine release syndrome (90), incorporating kill switches in the CAR construct may be needed in veterinary medicine as well.

### 4.2. Natural killer cells

NK cells have reduced risk of inducing a graft vs. host reponse and have shown promise in human preclinical studies. Moreover, human NK cells can be sourced allogeneically (91, 92), and infused at higher doses (93, 94). Allogeneic sourcing may allow mass production of an “off the shelf” product, reducing manufacturing costs, which is a significant concern in veterinary medicine.

Major challenges to using NK cells for veterinary CAR therapy include the lack of consensus on surface markers, limited antibody reagents, and lack of robust purification and expansion protocols. Feline NK cells are CD56^+^CD3^−^ (53, 59) and feline CD3 and CD56 antibodies exist (clones NZM1 and SZK1, respectively) (95, 96). However, there is not a consensus on canine NK markers. NKp46 is a common NK marker across species and CD3^−^NKp46^+^ cells enriched by FACS from canine peripheral blood mononuclear cells (PBMCs) exhibited cytotoxicity toward canine osteosarcoma and canine thyroid adenocarcinoma targets (60). Coculture of canine PBMCs with K562 cells expressing membrane bound IL15 and 4-1BBL, and added human IL2 and IL15, expanded large granular lymphocytes with cytotoxic activity (61). These presumptive NK cells were CD5^dim^CD3^+^CD8^+^TCRαβ^−^TCRγδ^−^CD21^−^CD4^−^ and although they did not have mRNA for CD56, they did have mRNAs for other NK receptors like NKG2D, NKp30, and NKp46. CD5 depleted canine PBMCs cultured with IL2 alone or IL2 and IL15 for 14 days also had NK-like cytotoxicity yet were CD56^−^ (97). CD94^+^ cells enriched from canine PBMCs were CD5^dim^NKp46^+^CD3^−^ (54). A first-in-canine clinical trial infused expanded cells with a similar phenotype into ten sarcoma patients in combination with intratumoral rhIL2 following focal radiotherapy (98). Five of the patients remained metastasis free at the 6-month primary endpoint (98). Despite NK cells being safe (99), their clinical efficacy does not yet match CAR T. Moreover, NK cells have a shorter *in vivo* lifespan than T cells. Addition of the *IL15* gene may provide sufficient signaling to overcome these limitations (100, 101).

### 4.3. Other cells

Immune cells such as natural killer T (NKT) cells, γδ T cells, and macrophages have been explored preclinically and clinically as human CAR drivers. Human NKT cells are rare CD3^+^ lymphocytes expressing an invariant αβ TCR, and may coexpress CD56 (65, 102, 103). Feline NKT cells are CD56^+^CD3^+^ (53, 59); however, canine NKT markers are more controversial. Originally defined as CD3^+^ lymphocytes that bound to complexes of α-galactosylceramide and murine CD1d (68), one group identified a CD5^intermediate^NKp46^+^CD94^+^CD3^+^ subset of large granular lymphocytes that may be NKT cells (54). Clinical isolation protocols for NKT cells may require dual CD56/CD3 enrichment for felines or NKp46/CD3 for canines, and there are currently no expansion protocols to obtain clinically useful numbers of these feline or canine cells. Regardless, human CD19 CAR NKT cells against lymphoma (104), and GD2 CAR NKT cells against neuroblastoma (105), have demonstrated preclinical efficacy, with CD19 CAR NKT cells exerting anti-lymphoma activity through both the CAR and the invariant TCR interaction with CD1d. However, not all tumors express CD1d and much of the activity will be *via* the CAR (106, 107). Human GD2 CAR NKT cells, co-expressing IL15, infused in pediatric neuroblastoma patients, were well-tolerated and reduced metastasis in one patient. This study provided safety data for human CAR NKT cells co-expressing self-supporting growth factors (108). NKT cells may soon be explored for CAR therapy in veterinary medicine.

In veterinary medicine, γδ T cells play an important role in mucosal immunity (109), and can comprise nearly half of the PBMC compartment in young ruminants (110). γδ T cells express TCRs with broad specificity and are MHC independent, yet they have *in vitro* cytotoxic activity similar to NK and T cells. Human GD2 CAR γδ T cells demonstrated *in vitro* cytotoxicity to the LAN1 neuroblastoma cell line (111). Both canine and feline *TCRG* loci have been identified and subsets can be classified through PCR, but robust isolation and expansion protocols are lacking (69, 70, 112). Moreover, many γδ T cells are located in peripheral tissues and may be difficult to enrich from peripheral blood in sufficient numbers to expand for clinical use (113). Enrichment of human Vδ1 cells from peripheral blood and expansion in cell culture bags using IFNγ, anti-CD3, and IL4, for 2 weeks followed by IL15 for 1 week, did generate a clinically relevant product yield and upregulation of effector markers (NKG2D, DNAM-1, NKp30, NKp44, and 2B4) (114). However, further research is needed to determine if γδ T cells will be useful in veterinary CAR therapy.

Macrophages are abundant in tumors of many different species, can exhibit anti-tumor activity, and have therapeutic potential as CAR drivers (115, 116). Macrophages can polarize to many different functional states from the extremes of proinflammatory M1 to anti-inflammatory/immunosuppressive M2 cells. Tumor-associated macrophages also adapt to the tumor microenvironment in ways that promote rather than eliminate tumors (117). In dogs, high numbers of macrophages in tumors is correlated with increased aggressiveness and worse prognosis for mammary cancer (72). Human THP-1 monocytic cells engineered to express CD19, HER2, or mesothelin CARs, phagocytosed target cells *in vitro* (118). Primary human HER2 CAR macrophages extended survival in a mouse ovarian xenograft model, suggesting that they still demonstrated antitumor activity despite the immunosuppressive tumor microenvironment (118). Macrophage immunotherapy in veterinary oncology has largely focused on *in vivo* activation of macrophages rather than *ex vivo* manipulation and reinfusion, but there is potential to develop them as CAR drivers (119–122), A limitation is that macrophages, and their precursor monocytes, are notoriously difficult to genetically modify regardless of species. Some approaches to overcome this limitation include using a replication-incompetent adenovirus (118, 123). Despite their limitations, macrophages and other CAR drivers warrant a basic science investigation to understand their true potential for use in veterinary medicine.

## 5. Discussion

Cell-based immunotherapy has gained traction as a promising therapeutic modality for multiple cancers in both human and veterinary patients. Although clinical veterinary studies are still in the beginning phases, the potential for breakthrough therapies, like has happened for human hematologic oncology, is high. Veterinary clinical trials involving infusions of T cells and NK cells have demonstrated the feasibility and safety of harvesting and manufacturing cells for clinical use (30, 78, 83, 98). However, to fully break into the cellular immunotherapy sector the way human medicine has, veterinary schools or other hospitals will need appropriate infrastructure for cellular manufacturing and genetic modification, or identify industry partners. Current manufacturing systems are designed for clinical production of human cellular therapeutics, but as interest in veterinary cell therapy grows, so will the market for xeno-free GMP-grade media, reagents, and supplements to be used for species-specific cell isolation and clinical expansion. The potential cost of the therapy also presents a major hurdle, and possibly the biggest challenge toward translation to clinical veterinary use. Insurance coverages that can defray the six-figure prices of human CAR T cell therapies would not be an option in veterinary medicine. Thus, a significant focus of future veterinary CAR research must be to develop more generally tolerable therapies with low levels of side effects to create a product that could be administered at a general veterinary practice. These will likely include a product where endogenous TCRs are deleted and other modifications are made to reduce cytokine release syndrome. Overall, companion animal patients may greatly benefit from immunotherapies that have seen success thus far in human patients due to their shared spontaneous disease development. As the field progresses in veterinary medicine, future treatment modalities designed for companion animals may one day translate back to human medicine.

## Author contributions

JC and CL conceived of the review, wrote, and edited the manuscript. JC performed searches in PubMed and Google Scholar databases including, but not limited to: CAR T therapy, chimeric antigen receptor, canine, feline, CAR NKT, macrophage markers, T cell phenotype, gamma delta T cell veterinary, and murine T cells. Both authors contributed to the article and approved the submitted version.

## References

[1] Deniger DC Pasetto A Tran E Parkhurst MR Cohen CJ Robbins PF. Stable, Nonviral expression of mutated tumor neoantigen-specific T-cell receptors using the sleeping beauty transposon/transposase system. Mol Ther. (2016) 24:1078–89. 10.1038/mt.2016.51 26945006 PMC4923320

[2] Jin J Sabatino M Somerville R Wilson JR Dudley ME Stroncek DF. Simplified method of the growth of human tumor infiltrating lymphocytes in gas-permeable flasks to numbers needed for patient treatment. J Immunother. (2012) 35:283–92. 10.1097/CJI.0b013e31824e801f 22421946 PMC3315105

[3] Dudley ME Wunderlich JR Shelton TE Even J Rosenberg SA. Generation of tumor-infiltrating lymphocyte cultures for use in adoptive transfer therapy for melanoma patients. J Immunother. (2003) 26:332–42. 10.1097/00002371-200307000-00005 12843795 PMC2305721

[4] Zacharakis N Chinnasamy H Black M Xu H Lu YC Zheng Z. Immune recognition of somatic mutations leading to complete durable regression in metastatic breast cancer. Nat Med. (2018) 24:724–30. 10.1038/s41591-018-0040-8 29867227 PMC6348479

[5] Rosenberg SA Yang JC Sherry RM Kammula US Hughes MS Phan GQ. Durable complete responses in heavily pretreated patients with metastatic melanoma using T-cell transfer immunotherapy. Clin Cancer Res. (2011) 17:4550–7. 10.1158/1078-0432.CCR-11-0116 21498393 PMC3131487

[6] Dudley ME. Cancer regression and autoimmunity in patients after clonal repopulation with antitumor lymphocytes. Science. (2002) 298:850–4. 10.1126/science.1076514 12242449 PMC1764179

[7] Berdeja JG Madduri D Usmani SZ Jakubowiak A Agha M Cohen AD. Ciltacabtagene autoleucel, a B-cell maturation antigen-directed chimeric antigen receptor T-cell therapy in patients with relapsed or refractory multiple myeloma (CARTITUDE-1): a phase 1b/2 open-label study. Lancet. (2021) 398:314–24. 10.1016/S0140-6736(21)00933-8 34175021

[8] Raje N Berdeja J Lin Y Siegel D Jagannath S Madduri D. Anti-BCMA CAR T-cell therapy bb2121 in relapsed or refractory multiple myeloma. N Engl J Med. (2019) 380:1726–37. 10.1056/NEJMoa1817226 31042825 PMC8202968

[9] Shah BD Bishop MR Oluwole OO Logan AC Baer MR Donnellan WB. KTE-X19 anti-CD19 CAR T-cell therapy in adult relapsed/refractory acute lymphoblastic leukemia: ZUMA-3 phase 1 results. Blood. (2021) 138:11–22. 10.1182/blood.2020009098 33827116 PMC9999039

[10] Abramson JS Palomba ML Gordon LI Lunning MA Wang M Arnason J. Lisocabtagene maraleucel for patients with relapsed or refractory large B-cell lymphomas (TRANSCEND NHL 001): a multicentre seamless design study. Lancet. (2020) 396:839–52. 10.1016/S0140-6736(20)31366-0 32888407

[11] Maude SL Laetsch TW Buechner J Rives S Boyer M Bittencourt H. Tisagenlecleucel in children and young adults with B-cell lymphoblastic leukemia. N Engl J Med. (2018) 378:439–48. 10.1056/NEJMoa1709866 29385370 PMC5996391

[12] Neelapu SS Locke FL Bartlett NL Lekakis LJ Miklos DB Jacobson CA. Axicabtagene ciloleucel CAR T-cell therapy in refractory large B-cell lymphoma. N Engl J Med. (2017) 377:2531–44. 10.1056/NEJMoa1707447 29226797 PMC5882485

[13] Vezzali E Parodi AL Marcato PS Bettini G. Histopathologic classification of 171 cases of canine and feline non-Hodgkin lymphoma according to the WHO. Vet Comp Oncol. (2010) 8:38–49. 10.1111/j.1476-5829.2009.00201.x 20230580

[14] Richards KL Suter SE. Man's best friend: what can pet dogs teach us about non-Hodgkin's lymphoma? Immunol Rev. (2015) 263:173–91. 10.1111/imr.12238 25510277 PMC4269254

[15] Jackson HJ Rafiq S Brentjens RJ. Driving CAR T-cells forward. Nat Rev Clin Oncol. (2016) 13:370–83. 10.1038/nrclinonc.2016.36 27000958 PMC5529102

[16] Sadelain M Brentjens R Rivière I. The basic principles of chimeric antigen receptor design. Cancer Discov. (2013) 3:388–98. 10.1158/2159-8290.CD-12-0548 23550147 PMC3667586

[17] Branella GM Spencer HT. Natural receptor- and ligand-based chimeric antigen receptors: strategies using natural ligands and receptors for targeted cell killing. Cells. (2021) 11:21. 10.3390/cells11010021 35011583 PMC8750724

[18] Fierle JK Abram-Saliba J Atsaves V Brioschi M de Tiani M Reichenbach P. A cell-based phenotypic library selection and screening approach for the de novo discovery of novel functional chimeric antigen receptors. Sci Rep. (2022) 12:1136. 10.1038/s41598-022-05058-5 35064152 PMC8782825

[19] Jensen MC Popplewell L Cooper LJ DiGiusto D Kalos M Ostberg JR. Antitransgene rejection responses contribute to attenuated persistence of adoptively transferred CD20/CD19-specific chimeric antigen receptor redirected T cells in humans. Biol Blood Marrow Transplant. (2010) 16:1245–56. 10.1016/j.bbmt.2010.03.014 20304086 PMC3383803

[20] Till BG Jensen MC Wang J Chen EY Wood BL Greisman HA. Adoptive immunotherapy for indolent non-Hodgkin lymphoma and mantle cell lymphoma using genetically modified autologous CD20-specific T cells. Blood. (2008) 112:2261–71. 10.1182/blood-2007-12-128843 18509084 PMC2532803

[21] Kawalekar OU O'Connor RS Fraietta JA Guo L McGettigan SE Posey AD. Distinct signaling of coreceptors regulates specific metabolism pathways and impacts memory development in CAR T cells. Immunity. (2016) 44:380–90. 10.1016/j.immuni.2016.01.021 26885860 PMC13498396

[22] Long AH Haso WM Shern JF Wanhainen KM Murgai M Ingaramo M. 4-1BB costimulation ameliorates T cell exhaustion induced by tonic signaling of chimeric antigen receptors. Nat Med. (2015) 21:581–90. 10.1038/nm.3838 25939063 PMC4458184

[23] Zhong XS Matsushita M Plotkin J Riviere I Sadelain M. Chimeric antigen receptors combining 4-1BB and CD28 signaling domains augment PI3kinase/AKT/Bcl-XL activation and CD8+ T cell–mediated tumor eradication. Mol Ther. (2010) 18:413–20. 10.1038/mt.2009.210 19773745 PMC2839303

[24] Wang J Jensen M Lin Y Sui X Chen E Lindgren CG. Optimizing adoptive polyclonal T cell immunotherapy of lymphomas, using a chimeric T cell receptor possessing CD28 and CD137 costimulatory domains. Hum Gene Ther. (2007) 18:712–25. 10.1089/hum.2007.028 17685852

[25] Mochel JP Ekker SC Johannes CM Jergens AE Allenspach K Bourgois-Mochel A. CAR T cell immunotherapy in human and veterinary oncology: changing the odds against hematological malignancies. AAPS J. (2019) 21:50. 10.1208/s12248-019-0322-1 30963322

[26] Levine BL Miskin J Wonnacott K Keir C. Global manufacturing of CAR T cell therapy. Mol Ther Methods Clin Dev. (2017) 4:92–101. 10.1016/j.omtm.2016.12.006 28344995 PMC5363291

[27] Levine BL. Performance-enhancing drugs: design and production of redirected chimeric antigen receptor (CAR) T cells. Cancer Gene Ther. (2015) 22:79–84. 10.1038/cgt.2015.5 25675873

[28] Guedan S Calderon H Posey AD Maus MV. Engineering and design of chimeric antigen receptors. Mol Ther Methods Clin Dev. (2019) 12:145–56. 10.1016/j.omtm.2018.12.009 30666307 PMC6330382

[29] Huang X Guo H Kang J Choi S Zhou TC Tammana S. Sleeping beauty transposon-mediated engineering of human primary T cells for therapy of CD19+ lymphoid malignancies. Mol Ther. (2008) 16:580–9. 10.1038/sj.mt.6300404 18227839 PMC4539139

[30] Panjwani MK Smith JB Schutsky K Gnanandarajah J O'Connor CM Powell DJ. Feasibility and safety of RNA-transfected CD20-specific chimeric antigen receptor T cells in dogs with spontaneous B cell lymphoma. Mol Ther. (2016) 24:1602–14. 10.1038/mt.2016.146 27401141 PMC5113111

[31] Beatty GL Haas AR Maus MV Torigian DA Soulen MC Plesa G. Mesothelin-specific chimeric antigen receptor mRNA-engineered T cells induce antitumor activity in solid malignancies. Cancer Immunol Res. (2014) 2:112–20. 10.1158/2326-6066.CIR-13-0170 24579088 PMC3932715

[32] Rurik JG Tombácz I Yadegari A Méndez Fernández PO Shewale SV Li L. CAR T cells produced *in vivo* to treat cardiac injury. Science. (2022) 375:91–6. 10.1126/science.abm0594 34990237 PMC9983611

[33] Sugita M Galetto R Zong H Ewing-Crystal N Trujillo-Alonso V Mencia-Trinchant N. Allogeneic TCRαβ deficient CAR T-cells targeting CD123 in acute myeloid leukemia. Nat Commun. (2022) 13:2227. 10.1038/s41467-022-29668-9 35484102 PMC9050731

[34] Georgiadis C Preece R Nickolay L Etuk A Petrova A Ladon D. Long terminal repeat CRISPR-CAR-coupled “universal” T cells mediate potent anti-leukemic effects. Mol Ther. (2018) 26:1215–27. 10.1016/j.ymthe.2018.02.025 29605708 PMC5993944

[35] Qasim W Zhan H Samarasinghe S Adams S Amrolia P Stafford S. Molecular remission of infant B-ALL after infusion of universal TALEN gene-edited CAR T cells. Sci Transl Med. (2017) 9:eaaj2013. 10.1126/scitranslmed.aaj2013 28123068

[36] Poirot L Philip B Schiffer-Mannioui C Le Clerre D Chion-Sotinel I Derniame S. Multiplex genome-edited T-cell manufacturing platform for “off-the-shelf” adoptive T-cell immunotherapies. Cancer Res. (2015) 75:3853–64. 10.1158/0008-5472.CAN-14-3321 26183927

[37] Eyquem J Mansilla-Soto J Giavridis T van der Stegen SJC Hamieh M Cunanan KM. Targeting a CAR to the TRAC locus with CRISPR/Cas9 enhances tumour rejection. Nature. (2017) 543:113–7. 10.1038/nature21405 28225754 PMC5558614

[38] Jo S Das S Williams A Chretien AS Pagliardini T Le Roy A. Endowing universal CAR T-cell with immune-evasive properties using TALEN-gene editing. Nat Commun. (2022) 13:3453. 10.1038/s41467-022-30896-2 35773273 PMC9247096

[39] Ren J Liu X Fang C Jiang S June CH Zhao Y. Multiplex genome editing to generate universal CAR T cells resistant to PD1 inhibition. Clin Cancer Res. (2017) 23:2255–66. 10.1158/1078-0432.CCR-16-1300 27815355 PMC5413401

[40] Alanis-Lobato G Zohren J McCarthy A Fogarty NME Kubikova N Hardman E. Frequent loss of heterozygosity in CRISPR-Cas9–edited early human embryos. Proc Natl Acad Sci USA. (2021) 118:e2004832117. 10.1073/pnas.2004832117 34050011 PMC8179174

[41] Zuccaro MV Xu J Mitchell C Marin D Zimmerman R Rana B. Allele-specific chromosome removal after Cas9 cleavage in human embryos. Cell. (2020) 183:1650–64.e15. 10.1016/j.cell.2020.10.025 33125898

[42] Skryabin BV Kummerfeld DM Gubar L Seeger B Kaiser H Stegemann A. Pervasive head-to-tail insertions of DNA templates mask desired CRISPR-Cas9–mediated genome editing events. Sci Adv. (2020) 6:eaax2941. 10.1126/sciadv.aax2941 32095517 PMC7015686

[43] Ottaviano G Georgiadis C Gkazi SA Syed F Zhan H Etuk A. Phase 1 clinical trial of CRISPR-engineered CAR19 universal T cells for treatment of children with refractory B cell leukemia. Sci Transl Med. (2022) 14:eabq3010. 10.1126/scitranslmed.abq3010 36288281

[44] Sakai O Yamamoto H Igase M Mizuno T. Optimization of culture conditions for the generation of canine CD20-CAR-T cells for adoptive immunotherapy. In Vivo. (2022) 36:764–72. 10.21873/invivo.12763 35241532 PMC8931891

[45] Addissie S Klingemann H. Cellular immunotherapy of canine cancer. Vet Sci. (2018) 5:100. 10.3390/vetsci5040100 30563208 PMC6313932

[46] Rotolo A Atherton MJ Kasper BT Haran KP Mason NJ. Genetic re-direction of canine primary T cells for clinical trial use in pet dogs with spontaneous cancer. STAR Protoc. (2021) 2:100905. 10.1016/j.xpro.2021.100905 34746864 PMC8551231

[47] Sakai O Igase M Mizuno T. Optimization of canine CD20 chimeric antigen receptor T cell manufacturing and *in vitro* cytotoxic activity against B-cell lymphoma. Vet Comp Oncol. (2020) 18:739–52. 10.1111/vco.12602 32329214

[48] Gagliardi C Khalil M Foster AE. Streamlined production of genetically modified T cells with activation, transduction and expansion in closed-system G-Rex bioreactors. Cytotherapy. (2019) 21:1246–57. 10.1016/j.jcyt.2019.10.006 31837737

[49] Davis BM Loghin ER Conway KR Zhang X. Automated closed-system expansion of pluripotent stem cell aggregates in a rocking-motion bioreactor. SLAS Technol. (2018) 23:364–73. 10.1177/2472630318760745 29481762

[50] Gee AP GMP CAR-T cell production. Best Pract Res Clin Haematol. (2018) 31:126–34. 10.1016/j.beha.2018.01.002 29909913

[51] Fraser AR Pass C Burgoyne P Atkinson A Bailey L Laurie A. Development, functional characterization and validation of methodology for GMP-compliant manufacture of phagocytic macrophages: a novel cellular therapeutic for liver cirrhosis. Cytotherapy. (2017) 19:1113–24. 10.1016/j.jcyt.2017.05.009 28673774 PMC5571439

[52] Granzin M Soltenborn S Müller S Kollet J Berg M Cerwenka A. Fully automated expansion and activation of clinical-grade natural killer cells for adoptive immunotherapy. Cytotherapy. (2015) 17:621–32. 10.1016/j.jcyt.2015.03.611 25881519 PMC8725994

[53] Vermeulen BL Devriendt B Olyslaegers DA Dedeurwaerder A Desmarets LM Grauwet KL. Natural killer cells: frequency, phenotype and function in healthy cats. Vet Immunol Immunopathol. (2012) 150:69–78. 10.1016/j.vetimm.2012.08.010 22985632

[54] Graves SS Gyurkocza B Stone DM Parker MH Abrams K Jochum C. Development and characterization of a canine-specific anti-CD94 (KLRD-1) monoclonal antibody. Vet Immunol Immunopathol. (2019) 211:10–8. 10.1016/j.vetimm.2019.03.005 31084888 PMC7048049

[55] Comazzi S Riondato F. Flow cytometry in the diagnosis of canine T-cell lymphoma. Front Vet Sci. (2021) 8:600963. 10.3389/fvets.2021.600963 33869314 PMC8044988

[56] Radtanakatikanon A Keller SM Darzentas N Moore PF Folch G Nguefack Ngoune V. Topology and expressed repertoire of the *Felis catus* T cell receptor loci. BMC Genomics. (2020) 21:20. 10.1186/s12864-019-6431-5 31906850 PMC6945721

[57] Vermeulen BL Devriendt B Olyslaegers DA Dedeurwaerder A Desmarets LM Favoreel HW. Suppression of NK cells and regulatory T lymphocytes in cats naturally infected with feline infectious peritonitis virus. Vet Microbiol. (2013) 164:46–59. 10.1016/j.vetmic.2013.01.042 23434014 PMC7117246

[58] Glusman G Rowen L Lee I Boysen C Roach JC Smit AFA. Comparative genomics of the human and mouse T cell receptor loci. Immunity. (2001) 15:337–49. 10.1016/S1074-7613(01)00200-X 11567625

[59] Simões RD Howard KE Dean GA. *In vivo* assessment of natural killer cell responses during chronic feline immunodeficiency virus infection. PLoS ONE. (2012) 7:e37606. 10.1371/journal.pone.0037606 22701523 PMC3365115

[60] Foltz JA Somanchi SS Yang Y Aquino-Lopez A Bishop EE Lee DA. NCR1 expression identifies canine natural killer cell subsets with phenotypic similarity to human natural killer cells. Front Immunol. (2016) 7:521. 10.3389/fimmu.2016.00521 27933061 PMC5120128

[61] Shin DJ Park JY Jang YY Lee JJ Lee YK Shin MG. *Ex vivo* expansion of canine cytotoxic large granular lymphocytes exhibiting characteristics of natural killer cells. Vet Immunol Immunopathol. (2013) 153:249–59. 10.1016/j.vetimm.2013.03.006 23548866 PMC3769186

[62] Deuse T Hu X Agbor-Enoh S Jang MK Alawi M Saygi C. The SIRPα-CD47 immune checkpoint in NK cells. J Exp Med. (2021) 218:e20200839. 10.1084/jem.20200839 33416832 PMC7802363

[63] Marquardt N Wilk E Pokoyski C Schmidt RE Jacobs R. Murine CXCR3 ^+^ CD27 ^bright^ NK cells resemble the human CD56 ^bright^ NK-cell population. Eur J Immunol. (2010) 40:1428–39. 10.1002/eji.200940056 20186880

[64] Milush JM Long BR Snyder-Cappione JE Cappione AJ York VA Ndhlovu LC. Functionally distinct subsets of human NK cells and monocyte/DC-like cells identified by coexpression of CD56, CD7, and CD4. Blood. (2009) 114:4823–31. 10.1182/blood-2009-04-216374 19805616 PMC2786291

[65] Krijgsman D de Vries NL Skovbo A Andersen MN Swets M Bastiaannet E. Characterization of circulating T-, NK-, and NKT cell subsets in patients with colorectal cancer: the peripheral blood immune cell profile. Cancer Immunol Immunother. (2019) 68:1011–24. 10.1007/s00262-019-02343-7 31053876 PMC6529387

[66] Hu Z Gu W Wei Y Liu G Wu S Liu T. Cells in mice originate from cytoplasmic CD3-positive, CD4–CD8– double-negative thymocytes that express CD44 and IL-7Rα. Sci Rep. (2019) 9:1874. 10.1038/s41598-018-37811-0 30755654 PMC6372634

[67] Johnston B Kim CH Soler D Emoto M Butcher EC. Differential chemokine responses and homing patterns of murine TCRαβ NKT cell subsets. J Immunol. (2003) 171:2960–9. 10.4049/jimmunol.171.6.2960 12960320

[68] Yasuda N Masuda K Tsukui T Teng A Ishii Y. Identification of canine natural CD3-positive T cells expressing an invariant T-cell receptor alpha chain. Vet Immunol Immunopathol. (2009) 132:224–31. 10.1016/j.vetimm.2009.08.002 19748683

[69] Keller SM Moore PF. A novel clonality assay for the assessment of canine T cell proliferations. Vet Immunol Immunopathol. (2012) 145:410–9. 10.1016/j.vetimm.2011.12.019 22237398

[70] Moore PF Woo JC Vernau W Kosten S Graham PS. Characterization of feline T cell receptor gamma (TCRG) variable region genes for the molecular diagnosis of feline intestinal T cell lymphoma. Vet Immunol Immunopathol. (2005) 106:167–78. 10.1016/j.vetimm.2005.02.014 15963816

[71] Siegers GM Swamy M Fernández-Malavé E Minguet S Rathmann S Guardo AC. Different composition of the human and the mouse γδ T cell receptor explains different phenotypes of CD3γ and CD3δ immunodeficiencies. J Exp Med. (2007) 204:2537–44. 10.1084/jem.20070782 17923503 PMC2118495

[72] Parisi F Tesi M Millanta F Gnocchi M Poli A. M1 and M2 tumour-associated macrophages subsets in canine malignant mammary tumours: an immunohistochemical study. Res Vet Sci. (2021) 136:32–8. 10.1016/j.rvsc.2021.02.007 33582312

[73] Ohara Y Yabuki A Nakamura R Ichii O Mizukawa H Yokoyama N. Renal infiltration of macrophages in canine and feline chronic kidney disease. J Comp Pathol. (2019) 170:53–9. 10.1016/j.jcpa.2019.05.006 31375159

[74] Vázquez S Vallejo R Espinosa J Arteche N Vega JA Pérez V. Immunohistochemical characterization of tumor-associated macrophages in canine lymphomas. Animals. (2021) 11:2301. 10.3390/ani11082301 34438760 PMC8388421

[75] Bertani FR Mozetic P Fioramonti M Iuliani M Ribelli G Pantano F. Classification of M1/M2-polarized human macrophages by label-free hyperspectral reflectance confocal microscopy and multivariate analysis. Sci Rep. (2017) 7:8965. 10.1038/s41598-017-08121-8 28827726 PMC5566322

[76] Jablonski KA Amici SA Webb LM Ruiz-Rosado JD Popovich PG Partida-Sanchez S. Novel markers to delineate murine M1 and M2 macrophages. PLoS ONE. (2015) 10:e0145342. 10.1371/journal.pone.0145342 26699615 PMC4689374

[77] Lloyd CM Phillips ARJ Cooper GJS Dunbar PR. Three-colour fluorescence immunohistochemistry reveals the diversity of cells staining for macrophage markers in murine spleen and liver. J Immunol Methods. (2008) 334:70–81. 10.1016/j.jim.2008.02.005 18367204

[78] Panjwani MK Atherton MJ MaloneyHuss MA Haran KP Xiong A Gupta M. Establishing a model system for evaluating CAR T cell therapy using dogs with spontaneous diffuse large B cell lymphoma. Oncoimmunology. (2020) 9:1676615. 10.1080/2162402X.2019.1676615 32002286 PMC6959441

[79] Wels W Harwerth IM Zwickl M Hardman N Groner B Hynes NE. Construction, bacterial expression and characterization of a bifunctional single–chain antibody–phosphatase fusion protein targeted to the human ERBB−2 receptor. Nat Biotechnol. (1992) 10:1128–32. 10.1038/nbt1092-1128 1369487

[80] Mata M Vera JF Gerken C Rooney CM Miller T Pfent C. Toward immunotherapy with redirected T cells in a large animal model: *ex vivo* activation, expansion, and genetic modification of canine T cells. J Immunother. (2014) 37:407–15. 10.1097/CJI.0000000000000052 25198528 PMC4163948

[81] Yin Y Boesteanu AC Binder ZA Xu C Reid RA Rodriguez JL. Checkpoint blockade reverses anergy in IL-13Rα2 humanized scFv-based CAR T cells to treat murine and canine gliomas. Mol Ther Oncolytics. (2018) 11:20–38. 10.1016/j.omto.2018.08.002 30306125 PMC6174845

[82] Loo D Alderson RF Chen FZ Huang L Zhang W Gorlatov S. Development of an Fc-enhanced anti–B7-H3 monoclonal antibody with potent antitumor activity. Clin Cancer Res. (2012) 18:3834–45. 10.1158/1078-0432.CCR-12-0715 22615450

[83] Zhang S Black RG Kohli K Hayes BJ Miller C Koehne A. B7-H3 specific CAR T cells for the naturally occurring, spontaneous canine sarcoma model. Mol Cancer Ther. (2022) 21:999–1009. 10.1158/1535-7163.MCT-21-0726 35405743 PMC9381119

[84] Davila ML Riviere I Wang X Bartido S Park J Curran K. Efficacy and toxicity management of 19-28z CAR T cell therapy in B cell acute lymphoblastic leukemia. Sci Transl Med. (2014) 6:224ra25. 10.1126/scitranslmed.3008226 24553386 PMC4684949

[85] Di Stasi A Tey SK Dotti G Fujita Y Kennedy-Nasser A Martinez C. Inducible apoptosis as a safety switch for adoptive cell therapy. N Engl J Med. (2011) 365:1673–83. 10.1056/NEJMoa1106152 22047558 PMC3236370

[86] Hoyos V Savoldo B Quintarelli C Mahendravada A Zhang M Vera J. Engineering CD19-specific T lymphocytes with interleukin-15 and a suicide gene to enhance their anti-lymphoma/leukemia effects and safety. Leukemia. (2010) 24:1160–70. 10.1038/leu.2010.75 20428207 PMC2888148

[87] Straathof KC Pulè MA Yotnda P Dotti G Vanin EF Brenner MK. An inducible caspase 9 safety switch for T-cell therapy. Blood. (2005) 105:4247–54. 10.1182/blood-2004-11-4564 15728125 PMC1895037

[88] Rivas C Miller AR Collado M Lam EW Apperley JF Melo JV. BCR-ABL-expressing cells transduced with the HSV-tk gene die by apoptosis upon treatment with ganciclovir. Mol Ther. (2001) 3:642–52. 10.1006/mthe.2001.0310 11356068

[89] Frank KB Chiou JF Cheng YC. Interaction of herpes simplex virus-induced DNA polymerase with 9-(1,3-dihydroxy-2-propoxymethyl)guanine triphosphate. J Biol Chem. (1984) 259:1566–9. 10.1016/S0021-9258(17)43446-6 6319402

[90] Atherton MJ Rotolo A Haran KP Mason NJ. Case report: clinical and serological hallmarks of cytokine release syndrome in a canine B cell lymphoma patient treated with autologous CAR-T cells. Front Vet Sci. (2022) 9:824982. 10.3389/fvets.2022.824982 35898541 PMC9310037

[91] Spanholtz J Preijers F Tordoir M Trilsbeek C Paardekooper J de Witte T. Clinical-grade generation of active NK cells from cord blood hematopoietic progenitor cells for immunotherapy using a closed-system culture process. PLoS ONE. (2011) 6:e20740. 10.1371/journal.pone.0020740 21698239 PMC3116834

[92] Li Y Schmidt-Wolf IGH Wu YF Huang SL Wei J Fang J. Optimized protocols for generation of cord blood-derived cytokine-induced killer/natural killer cells. Anticancer Res. (2010) 30:3493–9. 20944128

[93] Rezvani K. Adoptive cell therapy using engineered natural killer cells. Bone Marrow Transplant. (2019) 54:785–8. 10.1038/s41409-019-0601-6 31431708 PMC7594488

[94] Bollino D Webb TJ. Chimeric antigen receptor–engineered natural killer and natural killer T cells for cancer immunotherapy. Transl Res. (2017) 187:32–43. 10.1016/j.trsl.2017.06.003 28651074 PMC5604792

[95] Nishimura Y Shimojima M Sato E Izumiya Y Tohya Y Mikami T. Downmodulation of CD3epsilon expression in CD8alpha+beta- T cells of feline immunodeficiency virus-infected cats. J Gen Virol. (2004) 85(Pt 9):2585–9. 10.1099/vir.0.80102-0 15302952

[96] Shimojima M Nishimura Y Miyazawa T Kato K Tohya Y Akashi H. CD56 expression in feline lymphoid cells. J Vet Med Sci. (2003) 65:769–73. 10.1292/jvms.65.769 12939502

[97] Michael HT Ito D McCullar V Zhang B Miller JS Modiano JF. Isolation and characterization of canine natural killer cells. Vet Immunol Immunopathol. (2013) 155:211–7. 10.1016/j.vetimm.2013.06.013 23876304 PMC4019669

[98] Canter RJ Grossenbacher SK Foltz JA Sturgill IR Park JS Luna JI. Radiotherapy enhances natural killer cell cytotoxicity and localization in pre-clinical canine sarcomas and first-in-dog clinical trial. J Immunother Cancer. (2017) 5:98. 10.1186/s40425-017-0305-7 29254507 PMC5735903

[99] Liu E Marin D Banerjee P Macapinlac HA Thompson P Basar R. Use of CAR-transduced natural killer cells in CD19-positive lymphoid tumors. N Engl J Med. (2020) 382:545–53. 10.1056/NEJMoa1910607 32023374 PMC7101242

[100] Liu E Tong Y Dotti G Shaim H Savoldo B Mukherjee M. Cord blood NK cells engineered to express IL-15 and a CD19-targeted CAR show long-term persistence and potent antitumor activity. Leukemia. (2018) 32:520–31. 10.1038/leu.2017.226 28725044 PMC6063081

[101] Imamura M Shook D Kamiya T Shimasaki N Chai SMH Coustan-Smith E. Autonomous growth and increased cytotoxicity of natural killer cells expressing membrane-bound interleukin-15. Blood. (2014) 124:1081–8. 10.1182/blood-2014-02-556837 25006133

[102] Bernin H Fehling H Marggraff C Tannich E Lotter H. The cytokine profile of human NKT cells and PBMCs is dependent on donor sex and stimulus. Med Microbiol Immunol. (2016) 205:321–32. 10.1007/s00430-016-0449-y 26895635 PMC4939169

[103] Bendelac A Savage PB Teyton L. The biology of NKT Cells. Annu Rev Immunol. (2007) 25:297–336. 10.1146/annurev.immunol.25.022106.141711 17150027

[104] Rotolo A Caputo VS Holubova M Baxan N Dubois O Chaudhry MS. Enhanced anti-lymphoma activity of CAR19-iNKT cells underpinned by dual CD19 and CD1d targeting. Cancer Cell. (2018) 34:596–610.e11. 10.1016/j.ccell.2018.08.017 30300581 PMC6179961

[105] Xu X Huang W Heczey A Liu D Guo L Wood M. NKT Cells coexpressing a GD2-specific chimeric antigen receptor and IL15 show enhanced *in vivo* persistence and antitumor activity against neuroblastoma. Clin Cancer Res. (2019) 25:7126–38. 10.1158/1078-0432.CCR-19-0421 31484667 PMC6891170

[106] Hara A Koyama-Nasu R Takami M Toyoda T Aoki T Ihara F. CD1d expression in glioblastoma is a promising target for NKT cell-based cancer immunotherapy. Cancer Immunol Immunother. (2021) 70:1239–54. 10.1007/s00262-020-02742-1 33128583 PMC8053161

[107] Chong TW Goh FY Sim MY Huang HH Thike DAA Lim WK. CD1d expression in renal cell carcinoma is associated with higher relapse rates, poorer cancer-specific and overall survival. J Clin Pathol. (2015) 68:200–5. 10.1136/jclinpath-2014-202735 25477528 PMC4345982

[108] Heczey A Courtney AN Montalbano A Robinson S Liu K Li M. Anti-GD2 CAR-NKT cells in patients with relapsed or refractory neuroblastoma: an interim analysis. Nat Med. (2020) 26:1686–90. 10.1038/s41591-020-1074-2 33046868

[109] Guerra-Maupome M Slate JR McGill JL. Gamma delta T cell function in ruminants. Vet Clin North Am Food Anim Pract. (2019) 35:453–69. 10.1016/j.cvfa.2019.08.001 31590897

[110] Hein WR Mackay CR. Prominence of γδ T cells in the ruminant immune system. Immunol Today. (1991) 12:30–4. 10.1016/0167-5699(91)90109-7 1826600

[111] Capsomidis A Benthall G Van Acker HH Fisher J Kramer AM Abeln Z. Chimeric antigen receptor-engineered human gamma delta T cells: enhanced cytotoxicity with retention of cross presentation. Mol Ther. (2018) 26:354–65. 10.1016/j.ymthe.2017.12.001 29310916 PMC5835118

[112] Massari S Bellahcene F Vaccarelli G Carelli G Mineccia M Lefranc MP. The deduced structure of the T cell receptor gamma locus in *Canis lupus* familiaris. Mol Immunol. (2009) 46:2728–36. 10.1016/j.molimm.2009.05.008 19539375

[113] Nielsen MM Witherden DA Havran WL. γδ T cells in homeostasis and host defence of epithelial barrier tissues. Nat Rev Immunol. (2017) 17:733–45. 10.1038/nri.2017.101 28920588 PMC5771804

[114] Almeida AR Correia DV Fernandes-Platzgummer A da Silva CL da Silva MG Anjos DR. Delta one T cells for immunotherapy of chronic lymphocytic leukemia: clinical-grade expansion/differentiation and preclinical proof of concept. Clin Cancer Res. (2016) 22:5795–804. 10.1158/1078-0432.CCR-16-0597 27307596

[115] Pathria P Louis TL Varner JA. Targeting tumor-associated macrophages in cancer. Trends Immunol. (2019) 40:310–27. 10.1016/j.it.2019.02.003 30890304

[116] Mosser DM Edwards JP. Exploring the full spectrum of macrophage activation. Nat Rev Immunol. (2008) 8:958–69. 10.1038/nri2448 19029990 PMC2724991

[117] Burger M Thiounn N Denzinger S Kondas J Benoit G Chapado MS. The application of adjuvant autologous antravesical macrophage cell therapy vs. BCG in non-muscle invasive bladder cancer: a multicenter, randomized trial. J Transl Med. (2010) 8:54. 10.1186/1479-5876-8-54 20529333 PMC2893125

[118] Klichinsky M Ruella M Shestova O Lu XM Best A Zeeman M. Human chimeric antigen receptor macrophages for cancer immunotherapy. Nat Biotechnol. (2020) 38:947–53. 10.1038/s41587-020-0462-y 32361713 PMC7883632

[119] Weiskopf K Anderson KL Ito D Schnorr PJ Tomiyasu H Ring AM. Eradication of canine diffuse large B-cell lymphoma in a murine xenograft model with CD47 blockade and Anti-CD20. Cancer Immunol Res. (2016) 4:1072–87. 10.1158/2326-6066.CIR-16-0105 27856424 PMC5454476

[120] Kurzman ID Shi F Vail DM MacEwen EG. *In vitro* and *in vivo* enhancement of canine pulmonary alveolar macrophage cytotoxic activity against canine osteosarcoma cells. Cancer Biother Radiopharm. (1999) 14:121–8. 10.1089/cbr.1999.14.121 10850295

[121] Hogge GS Burkholder JK Culp J Albertini MR Dubielzig RR Keller ET. Development of human granulocyte-macrophage colony-stimulating factor-transfected tumor cell vaccines for the treatment of spontaneous canine cancer. Hum Gene Ther. (1998) 9:1851–61. 10.1089/hum.1998.9.13-1851 9741424

[122] Fox LE MacEwen EG Kurzman RD Dubielzig RR Helfand SC Vail DM. Liposome-encapsulated muramyl tripeptide phosphatidylethanolamine for the treatment of feline mammary adenocarcinoma-a multicenter randomized double-blind study. Cancer Biother. (1995) 10:125–30. 10.1089/cbr.1995.10.125 7663571

[123] Nilsson M Ljungberg J Richter J Kiefer T Magnusson M Lieber A. Development of an adenoviral vector system with adenovirus serotype 35 tropism; efficient transient gene transfer into primary malignant hematopoietic cells. J Gene Med. (2004) 6:631–41. 10.1002/jgm.543 15170734

